# Comparative Efficacy of Clinic-Based and Telerehabilitation Application of Mckenzie Therapy in Chronic Low-Back Pain

**DOI:** 10.5195/ijt.2019.6260

**Published:** 2019-06-12

**Authors:** CHIDOZIE E. MBADA, MISTURA I. OLAOYE, OLUMIDE O. DADA, OLUSOLA AYANNIYI, OLUBUSOLA E. JOHNSON, ADESOLA C. ODOLE, GAMBO P. ISHAYA, OLUWATOSIN J. OMOLE, MOSES O. MAKINDE

**Affiliations:** 1 DEPARTMENT OF MEDICAL REHABILITATION, COLLEGE OF HEALTH SCIENCES, OBAFEMI AWOLOWO UNIVERSITY, ILE – IFE, NIGERIA; 2 DEPARTMENT OF PHYSIOTHERAPY, FACULTY OF ALLIED HEALTH SCIENCES, UNIVERSITY OF MEDICAL SCIENCES, ONDO STATE, NIGERIA; 3 DEPARTMENT OF PHYSIOTHERAPY, FACULTY OF CLINICAL SCIENCES, COLLEGE OF MEDICINE, UNIVERSITY OF IBADAN, NIGERIA; 4 DEPARTMENT OF COMPUTER SCIENCE AND ENGINEERING, FACULTY OF TECHNOLOGY, OBAFEMI AWOLOWO UNIVERSITY, ILE-IFE, NIGERIA

**Keywords:** Low-Back Pain, McKenzie Therapy, Mobile-App, Nigeria, Telerehabilitation

## Abstract

Studies on validation of telerehabilitation as an effective platform to help manage as well as reduce burden of care for Low-Back Pain (LBP) are sparse. This study compared the effects of Telerehabilitation-Based McKenzie Therapy (TBMT) and Clinic-Based McKenzie Therapy (CBMT) among patients with LBP. Forty-seven consenting patients with chronic LBP who demonstrated ‘directional preference’ for McKenzie Extension Protocol (MEP) completed this quasi experimental study. The participants were assigned into either the CBMT or TBMT group using block permuted randomization. Participants in the CBMT and TBMT groups received MEP involving a specific sequence of lumbosacral repeated movements in extension aimed to centralize, decrease, or abolish symptoms, thrice weekly for eight weeks. TBMT is a comparable version of CBMT performed in the home with the assistance of a mobile phone app. Outcomes were assessed at the 4th and 8th weeks of the study in terms of Pain Intensity (PI), Back Extensors Muscles’ Endurance (BEME), Activity Limitation (AL), Participation Restriction (PR), and General Health Status (GHS). Data were analyzed using descriptive and inferential statistics. Alpha level was set at p< 0.05. Within-group comparison across baseline, 4^th^ and 8^th^ weeks indicate that both CBMT and TBMT had significant effects on PI (p=0.001), BEME (p=0.001), AL (p=0.001), PR (p=0.001) and GHS (p=0.001) respectively. However, there were no significant differences (p>0.05) in the treatment effects between TBMT and CBMT, except for ‘vitality’ (p=0.011) scale in the GHS where TBMT led to significantly higher mean score. Mobile-app platform of the McKenzie extension protocol has comparable clinical outcomes with the traditional clinic-based McKenzie Therapy, and thus is an effective supplementary platform for care of patients with low-back pain.

Chronic Low-Back Pain (LBP) is more resistant to therapeutic intervention than the other forms of back pain (Fersum 2010), as a result, treatment intervention in the affected patients give variable outcomes (Rainville, Sobel, Hartigan, & Wright, 1997). Nonetheless, there is still evidence from randomized controlled trials that multidisciplinary programs, behavioural therapy and exercise are effective for chronic LBP (van Middelkoop et al., 2011). Similarly, systematic reviews of evidence concerning exercises concluded that exercises may be helpful for patients with chronic LBP, especially in terms of decrease in pain and disability (Hayden, van Tulder, Malmivaara, & Koes, 2005), decrease in fear of avoidance behaviour (van Tulder, Koes, & Bouter, 1997) and return to normal activities of daily living and work (Staalet et al., 2002).

Consequent to the foregoing, exercise has become the central element in the physical therapy management of patients with chronic non-specific LBP (Hayden, van Tulder, Malmivaara, & Koes, 2005; van Tulder et al., 2003). Still, the most effective exercise design to attain maximum benefits remains a subject of debate and continuous research (Taimela, Diederich, Hubsch, & Heinricy, 2000). The sub-grouping of patients with LBP according to their signs and symptoms as a prerequisite for exercise prescription is considered an important advance in the management of LBP (Long, Donelson, & Fung, 2004). One of the more commonly used methods of sub-grouping patients for intervention amongst physical therapists is the McKenzie method (McKenzie & May, 2003).

The McKenzie method is a classification-based treatment for LBP (Foster, Thompson, Baxter, Allen, 1999) with substantial evidence on its effectiveness (Machado, De Souza, Ferreira, & Ferreira, 2006; Nwuga & Nwuga, 1985). The McKenzie method sub-grouping is based on the patient’s directional preference. Directional preference is defined as the movement or posture that decreases or centralizes pain that emanates from the spine and/or increases range of movement (McKenzie & May, 2003). However, the strong association between having higher training in McKenzie therapy by physical therapists and obtaining positive therapeutic outcomes (Mooney, 1995) is a potential limitation in providing access to the McKenzie Therapy (MT), especially where there are no certified faculties.

Telerehabilitation is considered as a potential solution to bridge service delivery gap, especially in geographically remote areas with shortage of health care personnel and lack of access to physical therapy rehabilitation services (Dansky, Palmer, Shea, & Bowles, 2001). Telerehabilitation is described as the remote conveyance of healthcare services and clinical information using information and telecommunication technologies involving internet, wireless satellite and telephone media to provide series of rehabilitation services by eliminating the barriers of distance, time and travel to receive care (American Telemedicine Association [ATA], 2013). With the advent of smartphones, there is an abundance of commercially available applications offered for health care monitoring and management (Vashist, Schneider, & Luong, 2014). However, one of the major shortcomings of existing apps is that they rarely adhere to established guidelines or link to scientifically proven concepts (Abroms, Padmanabhan, Thaweethai, & Phillips, 2011; Huckvale, Car, Morrison, & Car, 2012). A number of studies have employed telerehabilitation methods with patients with LBP, mainly for assessment (Palacín-Marín et al., 2013; Truter, Russell, & Fary, 2014). However, there is an apparent dearth of studies on the telerehabilitation application of McKenzie therapy in patients with chronic non-specific LBP. The objective of this study was to compare the efficacy of Clinic-Based MT (CBMT) and Telerehabilitation-Based McKenzie Therapy (TBMT) on physiological (pain intensity and back muscles endurance) and psychosocial (activity limitation, participation restriction, and general health status) variables in patients with chronic non-specific LBP.

## MATERIALS AND METHODS

**Figure 1:**
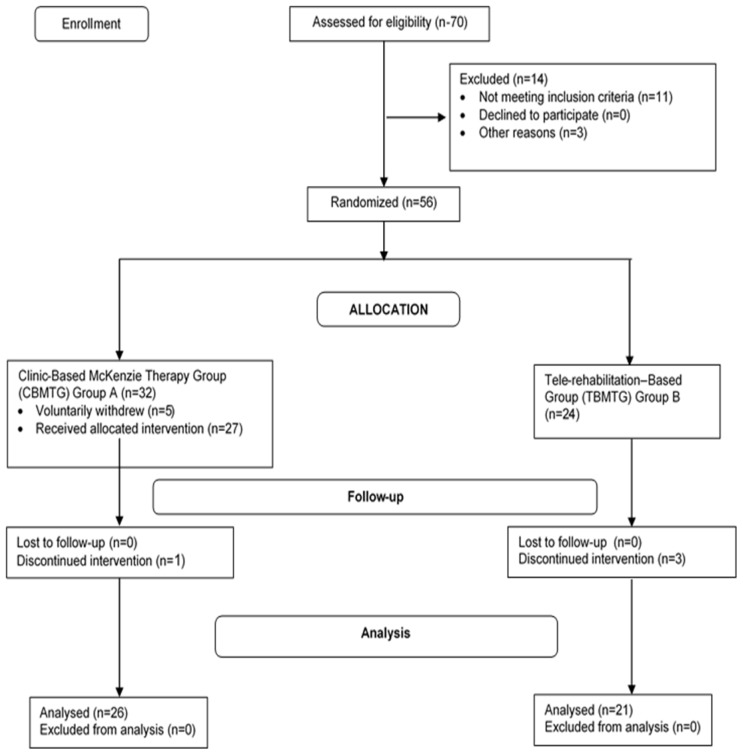
CONSORT showing the progression of patients through the study.

Seventy consecutive patients with chronic non-specific LBP attending the outpatient Physiotherapy Departments of the Ladoke Akintola University of Technology University Teaching Hospital (LAUTECH), Osogbo and the State Hospital, Ejigbo were invited into this quasi-experimental study. However, only 56 of the consenting patients were found eligible for the study, and 47 completed the study. CONSORT showing the progression of patients through the study is presented in Figure 1. Eligible participants for the study were patients with clinical diagnosis of chronic non-specific LBP who were between the ages of 20 and 65 years, and those without any obvious deformities affecting the trunk or upper and lower extremities. Exclusion criteria for this study included having a known co-morbidity or reported history of cardiovascular disease contraindicating exercise; being pregnant; previous back surgery; previous experience of the McKenzie therapy; and having directional preference for flexion or no directional preference based on the McKenzie Assessment. Sample size estimation for the study was based on the equation c ×π1 (1-π1) + π2 (1-π2)/(π1 − π2)^2^ (Chan, 2003), where c = 7.9 for 80% power, and π 1 and π2 are proportion estimates (π1 = 0.25 and π2 = 0.65). Therefore, n = 7.9 * (0.25 (1 – 0.25) + 0.65 (1 – 0.65)/(0.25 – 0.65) = 20.49 which is approximately 21. Hence, calculated N was 42 (21 per group). In order to account for 10% possible attrition (i.e., 4.2), the estimated minimum sample size was 46.

### INSTRUMENT

The following instruments were used in this study

- Quadruple Visual Analogue Scale (QVAS): This was used to assess pain intensity experienced by the participants at the time of assessment, typical or average pain, pain at its best, and pain at its worst, respectively (Von Korff, Le Resche & Dworkin, 1993). A Yoruba translated version of the QVAS was used for participants who had preference for the Yoruba language. The Yoruba version of the QVAS has a reliability co-efficient of r = 0.88.
- Oswestry Disability Index (ODI): This was used to assess participation restriction (Fairbank, Couper, Davies, & O’Brien, 1980). A Yoruba translated version of the ODI was also used in the study, and has a correlation coefficient (r) of 0.86.
- Roland Morris Disability Questionnaire (RMDQ): This was used to assess activity limitation in activity of daily living among the participants. Similarly, a Yoruba translated version of the 24 item RMDQ (Mbada et al., 2017) was used in the study.
- SF-12 General Health Status Questionnaire: This was used to assess the general health status (GHS) or the Health-related Quality of Life (HRQoL) of the participants. A Yoruba version of the SF-12 was also used in this study (2015).

All translations of the tools used in this study were done by language experts from the Department of Linguistics and African Languages, Obafemi Awolowo University, Ile Ife, Nigeria.

### PROCEDURE

Ethical approval for this study was obtained from the Health Research Ethical Committee of the Institute of Public Health, Obafemi Awolowo University Research and Ethical Committee before the commencement of the study. The purpose of the research was explained to each consenting participant. A Yoruba-translated informed consent form was also used in the study. Participants were consecutively recruited but randomly assigned to the two treatment groups until they had all completed the 8-week treatment program. In order to introduce blinding and reduce bias, a research assistant recorded the number of patients who were invited to participate, the number who declined to participate, and the number of screened patients who were ineligible and their reasons for declining participation or ineligibility. Participants who volunteered to participate and satisfied the eligibility criteria were randomly allocated to group A or B by the same assistant who was not involved in the assessment and treatment of the patient. In order to ensure equal-sized treatment groups, random permuted blocks were used (Pocock, 1979) and a block size of four was chosen (i.e., AABB, ABAB and all the other possible restricted permutations). The block permutations were computer-generated using a factorial equation formula: as “4!“ which is 1×2×3×4 = 24. The printout of all the 24 restricted computer-generated block permutation was sequentially numbered, cut, and placed in sealed envelope. Block permuted sequence was randomly drawn from the envelope and accordingly, consecutive patients were assigned to either the CBMT (i.e., group A) or the TBMT (i.e., group B). The process of drawing block permuted sequence and randomization was repeated as participants volunteered for the study.

### PRE-TREATMENT SCREENING

Baseline assessment was carried out for each participant in the study. Anthropometric variables involving weight and height were measured. The participants were screened for their eligibility to participate in the study using the McKenzie Institute’s Lumbar Spine Assessment Algorithm (MILSAA). The MILSAA sought information on demographics and LBP-specific characteristics including onset of back pain, recurrence, duration of complaint, and previous intervention received. The MILSAA is a well-defined algorithm that leads to the simple classification of spinal-related disorders. This is based on a consistent “cause and effect” relationship between historical pain behaviour as well as the pain response to repeated test movements, positions, and activities during the assessment process. The participants were assessed for directional preference. This involved repeated movements, between 5–10 sets of each movement and included movements in standing and lying and in sagittal and frontal planes while the participants’ symptomatic and mechanical responses were assessed. Following the repeated-movement testing, the participants returned to the same standing position and following standardized instructions in the MILSAA, they were asked whether pain was centralizing or peripheralizing during and after movements, or if there was no effect. The participants’ mechanical responses to repeated movements was used to establish their directional preferences. Flexion, lateral, and no responders to repeated movements were excluded from the study. Only extension responders from the MILSAA assessment were eligible for the study. None of the participants reported positively to the specific questions in the MILSAA indicative of red flags. None of the participants reported a current episode of constant symptoms of LBP. Among all the participants, test movements in flexion in standing produced pain, while repeated movements in flexion increased pain in either standing or lying. On the other hand, test movements and repeated movements in extension in standing and/or lying decreased and/or centralized pain among the participants. Following their qualification to participate in the study, each participant was requested to complete the outcome measures before the commencement of the exercise protocol and subsequently at weeks 4 and 8 of the study.

### PHYSICAL PERFORMANCE TEST

Static back extensors endurance was conducted prior the commencement of intervention using the modified Biering-Sørensen test of Static Muscular Endurance (BSME). The BSME was preceded by a warm-up phase of low-intensity self-pace walking and active stretching of the trunk and the extremities for about five minutes. During the BSME, the participant lay on a plinth in prone position with the upper edge of the iliac crests aligned with the edge of the plinth, and with hands held by the sides. The lower body (the lower extremities) was fixed to the plinth by two non-elastic straps located around the pelvis and ankles. Horizontality in the test position was ensured by asking the participant to maintain contact between his/her back and a hanging ball. Once a loss of contact for more than 10 seconds was noticed, the participant was encouraged once to immediately maintain contact again. Once the participant could not immediately correct or hold the position or claimed to be fatigued, the test was terminated (Biering-Sorensen, 1984; Mbada, Ayanniyi & Adedoyin, 2009).

## INTERVENTION

### CLINIC-BASED MCKENZIE THERAPY

The CBMT group received the McKenzie extension protocol. The protocol involves a course of specific lumbosacral repeated movements in extension that cause the symptoms to centralize, decrease, or abolish (McKenzie, 1990). The extension activities include Extension Lying Prone, Extension in Prone, and Extension in Standing, repeated up to ten times (McKenzie, 1990; McKenzie & May, 2003). The determination of the directional preference for extension was followed by the extension protocol. Detail of the protocol has been described in an earlier publication (Mbada et al., 2015). In addition to McKenzie extension protocol, the CBMT received a set of back care education instructions comprised of a 9-item instructional guide on standing, sitting, lifting, and other activities of daily living for home (McKenzie, 1990). A Yoruba version of the back education pamphlet was made available for participants who were literate in or preferred the Yoruba language.

### TELEREHABILITATION-BASED MCKENZIE THERAPY

Telerehabilitation-based McKenzie therapy is a comparable version of CBMT performed in the home with the assistance of a mobile phone app. The TBMT app is a combination of the McKenzie extension protocols and back care education developed and enabled to run on a smartphone or android phone with Operating System of 3.0. The app is exclusively a product of the authors and not that of the McKenzie Institute International. It incorporated personalized and guided self-therapy using the same protocols in the McKenzie protocol (i.e., Extension Lying Prone, Extension in Prone, and Extension in Standing). Thus, the TBMT is a mobile phone video app designed for patients with chronic LBP based on McKenzie therapy principles.

**Figure 2:**
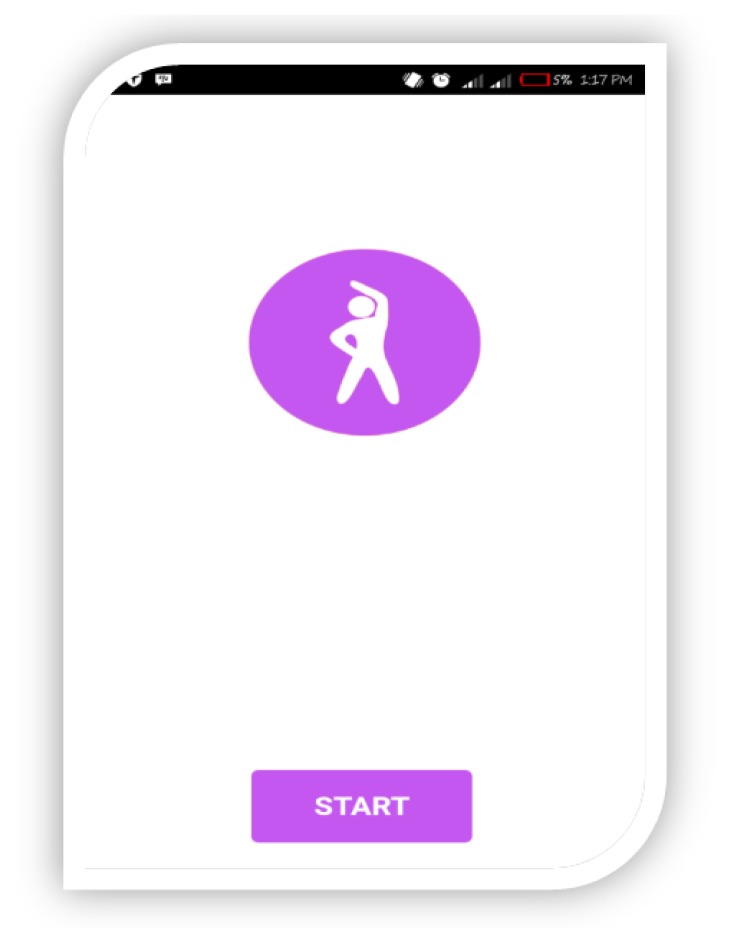
User Interface skin of the TBMT app.

**Figure 3:**
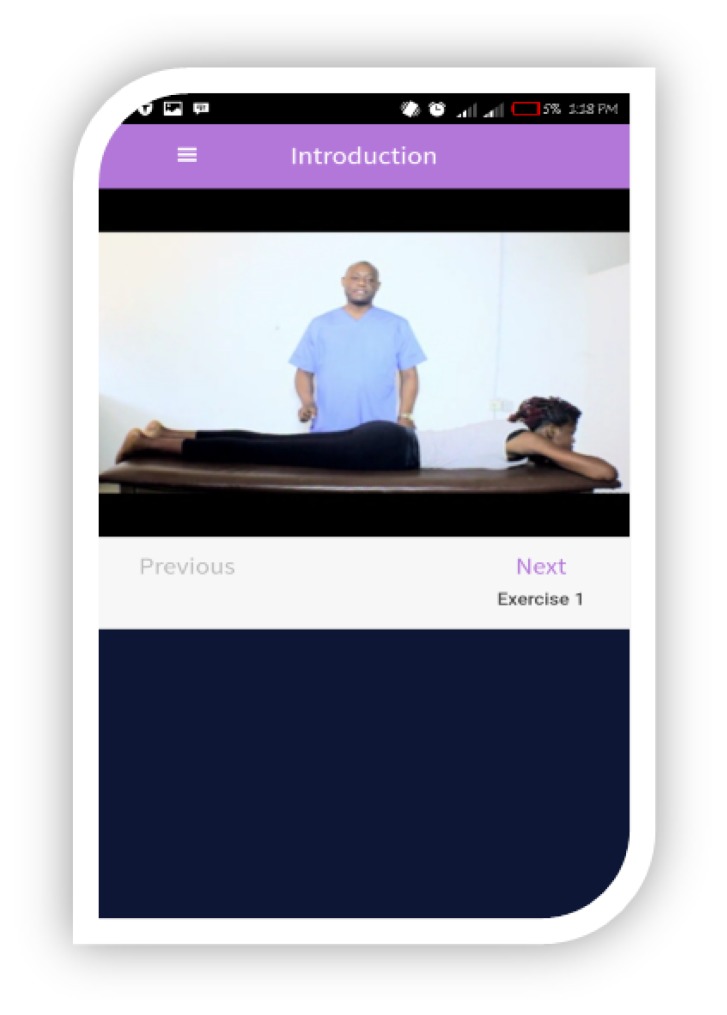
McKenzie extension exercise start position.

**Figure 4:**
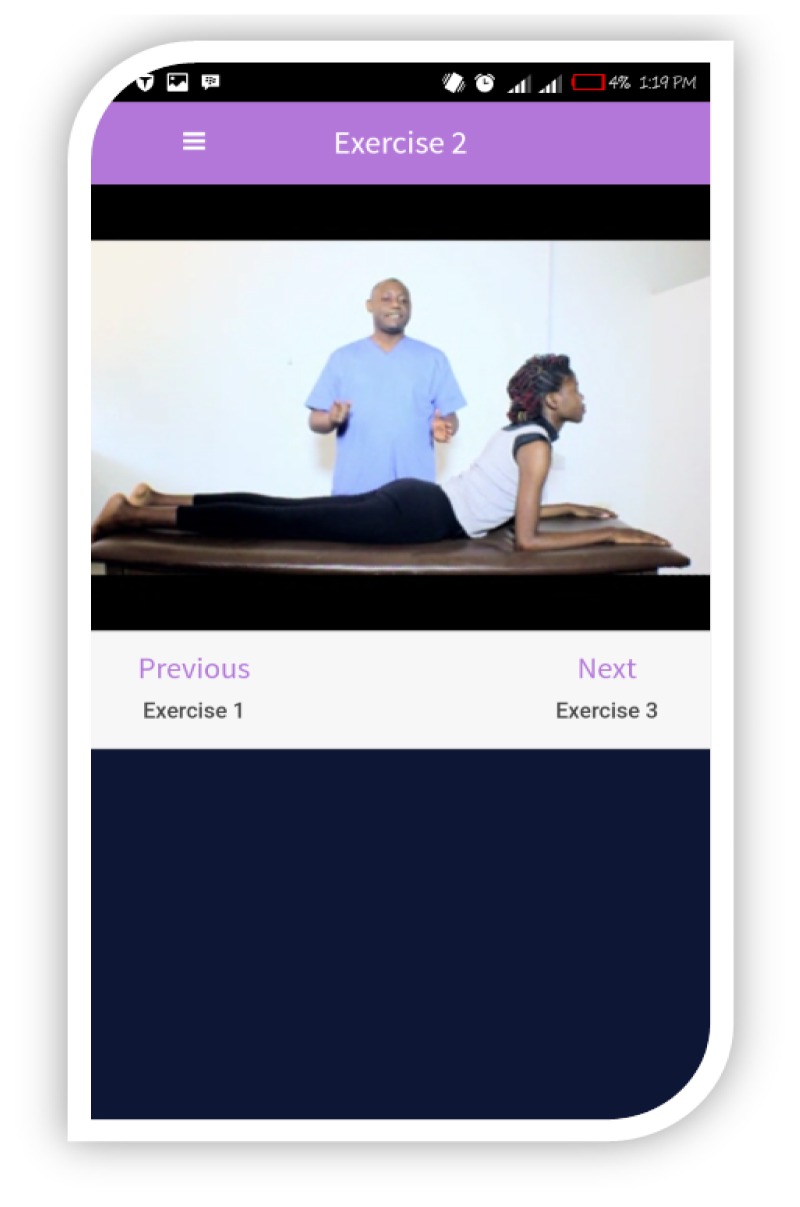
McKenzie extension exercise 2.

**Figure 5:**
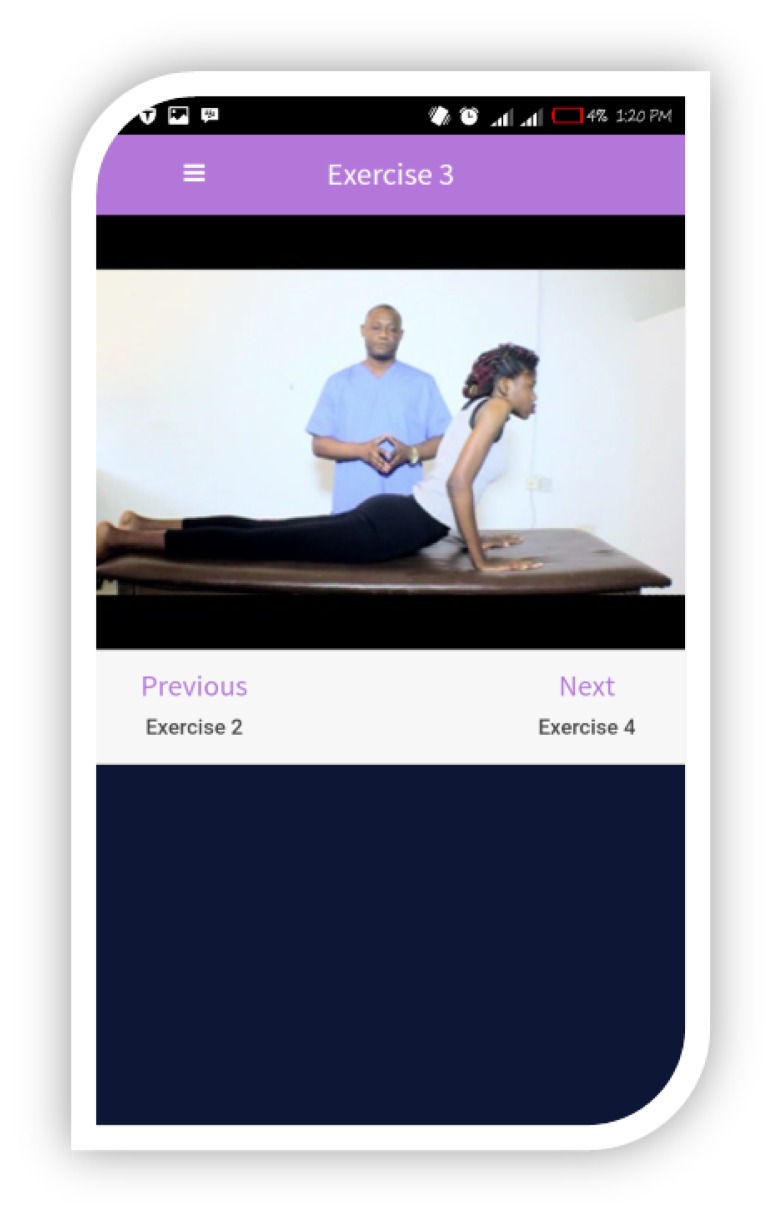
McKenzie extension exercise 3.

The app has moderate to high usability and functionality features based on the findings on its development and feasibility (Mbada et al., 2018). This functional app has a customized user interface skin and cycle feedback. The videos are preceded by an introduction, followed by four short exercises. Exercises 1 to 3 are graded extension activities in prone lying, while exercise 4 is comprised of extension activities in standing. The exercises are proceeded by back hygiene instructions. The app has features that allow users to pause, revert, or proceed to the next exercise. The app total run time is approximately five minutes. Figures 2 to 5 show some of the interface features of the TBMT app (Mbada et al., 2018). Adherence and utilization tracking of TBMT app was tele-monitored through phone calls and SMSs to the participants, in some instances, to their caregivers in order to guarantee engagement and therapy compliance.

### OUTCOME ASSESSMENT

Treatment outcome assessments were carried out at the 4^th^ and 8^th^ weeks of the study. During these assessment sessions, participants underwent the modified BSME, as well as, completed all the outcome tools. All outcome assessments were done in the clinic.

### DATA ANALYSIS

Descriptive statistics of mean and standard deviation were used to summarize the data. Comparing between the two groups, independent t-tests were used to compare demographic characteristics and patients outcomes that were continuous variables (QVAS, RMLDQ and BSME scores), while Mann Whitney U-tests were used for the categorical variables (ODI and SF-12 scores). For within groups effects, repeated measure ANOVA was used to determine the effects of the different treatment regimen across baseline, 4^th^ and 8^th^ week for continuous variables, while Friedman’s ANOVA was used for categorical variables. Alpha level was set at 0.05. The data analysis was carried out using IBM Statistics SPSS 22.0 version software (SPSS Inc., Chicago, Illinois, USA).

## RESULTS

Participants in the two groups were comparable in general characteristics (p>0.05) (Table 1). A majority of these participants in both groups were females (CBMT =76.9%; TBMT = 66.7%) (Table 2). While, half (50.0%) and two-thirds (66.7%) of the participants in CBMT and TBMT had previous history of LBP, most had no reduced range of motion (CBMT =88.5%; TBMT = 95.2%). Poor posture was the most implicated cause of LBP among the participants (CBMT 46.1%; TBMT = 42.9%). The most reported aggravating and alleviating factors for LBP were bending (CBMT =34.6%; TBMT = 28.5%) and lying (CBMT =61.5%; TBMT = 38.1%) (Table 2), and LBP was reported to mostly disturb sleep in the CBMT group (n=50%).

Table 3 shows comparison of participants’ baseline measures. Both groups were comparable in all measures (p>0.05), except the vitality scale of the SF-12. Tables 4 and 5 show the effect of each intervention across baseline, 4^th^ and 8^th^ week. Results shows that there were significant differences (p<0.05) in the outcome parameters across the three time points of the study.

Between groups comparison of effects showed no significant differences (p>0.05) in the treatment outcome (mean change) at the end of the 4^th^ week of the study (i.e., difference between baseline and week 4 values) (Table 6). Similarly, there were no significant differences (p>0.05) in the treatment outcome (mean change) across the two groups at the end of the 8^th^ week of the study (i.e., difference between baseline and week 4 values), except for items ‘vitality’ (p=0.011) on the SF-12 where the TBMT had significantly higher mean change (Table 7).

## DISCUSSION

This study compared the effect of CBMT and TBMT on pain intensity, back extensors muscles’ endurance, activity limitation, participation restriction, general health status, and cost-utility in patients with chronic non-specific LBP. The participants in this study were on the average 48.8±11.1years old. The age category of participants in this study fell within the age bracket of 40–80 years within which LBP is prevalent (Hoy et al., 2012). Also, more women than men were available to be recruited into this study. This finding is in keeping with the perception that women often report for pain from the musculoskeletal system than men (Stenberg & Ahlgren, 2010), and also reinforced the common report that women display a greater willingness to seek care for health issues (Hunt, Adamson, Hewitt, & Nazareth, 2011). Accordingly, Hoy et al. (2012) reported that LBP is more prevalent among female individuals.

The clinical characteristics profile of the patients in this study, showed that the patients’ pain was longstanding (about nine months), with the majority having recurrent episodes of LBP. LBP is reported to run a recurrent course in the majority of patients (Carey, Garrett, Jackman, & Hadler, 1999). In essence, it implies that following an episode of LBP, it is likely that a patient will have further episodes of pain causing suffering for the patient and time loss from work (Stanton et al., 2008). However, the area of recurrent LBP is complex (Stanton, Latimer, Maher, & Hancock, 2010). Furthermore, patients in this study implicated poor posture as the main cause of their LBP episodes. This report is consistent with literature showing that poor posture is a major etiology for LBP. However, the link between spinal posture and LBP is still contestable (Mitchell et al., 2008). Studies have found strong associations between LBP and positions of the lumbar spine in flexion and rotation (Heneweer et al., 2011). Compared to standing posture, sitting posture decreases lumbar lordosis and increases low back muscle activity, disc pressure, and pressure on the ischium which are associated with the development of LBP. Sitting with reduced ischial support and a fitted backrest reduces the low back muscle activity which also increases sitting comfort and reduces the risk of development of LBP (Makhsous et al., 2003). Heavy physical loading, trauma, poor and prolonged postures in bending, twisting, and non-neutral work positions coupled with carrying heavy physical loads have been associated with disc degeneration (Omair et al., 2013). The findings of this study also showed that the most aggravating and alleviating factors for LBP were bending and lying.

There were reports that LBP disturbed sleep among some patients in this study, consistent with the findings of prior studies that chronic LBP significantly affects quality of sleep (Alsaadi et al., 2011; Marin, Cyhan & Miklos, 2006). It has been suggested that sleep problems should be addressed as an integral part of the pain management plan (Alsaadi et al., 2011; Marin, Cyhan, & Miklos, 2006).

None of the patients in this study reported positively to specific questions in the McKenzie Institute Lumbar Spine Assessment Algorithm that were indicative of red flags. Also, none of the patients reported a current episode of constant symptoms of LBP. For all participants, test movements in flexion in standing produced pain, while repeated movements in flexion increased pain in either standing or lying. On the other hand, test movements and repeated movements in extension in standing and/or lying decreased and/or centralized pain among the participants.

The patients in this study were comparable in their general characteristics, except for weight. Similarly, their baseline clinical measures were comparable, except for ‘vitality’ on the SF-12 health survey. Baseline characteristics are believed to be predictors of response to treatment in clinical trials for LBP (Underwood, Morton, Farrin, & UK BEAM Trial Team, 2007). Hence, comparability in baseline measures in clinical trials is reported to reduce the chances of confounders other than the intervention in predicting outcomes. However, Friedman, Furberg, and DeMets (2010) submit that for many measurements, baseline data may not reflect a participant’s true condition at the time of baseline, because investigators perform baseline assessment close to the time of intervention. Therefore, the results obtained at different points in the course of this study could have been largely due to the effects of the various treatment regimens.

The within-group comparison of participants in CBMT and TBMT groups across the three time points of the study revealed that McKenzie extension therapy plus back hygiene conducted via the traditional clinic-based approach or performed in the home with the assistance of a mobile phone app had significant effects on pain intensity, back extensors muscles’ endurance, activity limitation, participation restriction, and general health status. These findings are consistent with previous reports that demonstrated evidence for use of the McKenzie protocol (Machado, De Souza, Ferreira, & Ferreira, 2006; Nwuga & Nwuga, 1985). Thus, irrespective of the mode of delivery, the McKenzie protocol seems to have significant effects in terms of all the treatment outcomes.

The mechanism by which the McKenzie protocol achieves its therapeutic effects is largely dependent on patients’ differences and pathologic conditions as per the type of McKenzie syndrome. For example, derangement syndrome is believed to result in obstructed range of motion (McKenzie, 1990). McKenzie postulated that spinal flexion causes a movement of the nucleus pulposus to a more posterior position due to increased mechanical compression on the anterior surface of the intervertebral disc (McKenzie, 1990). Therefore, extension in derangement syndrome is proposed to help alleviate stress on the posterior annulus, decrease nerve root compression, and thereby relieve pain (Ordway et al., 1999). Nuclear pressure is reduced when compressive force is transferred from the vertebral disc body unit to the apophyseal joints during extension exercise (Quinnell, Stockdale, & Willis, 1983). Furthermore, Adams et al. (2000) posits that the posterior annulus can be stress shielded by the neural arch in extended postures, and this may explain why extension exercises can relieve LBP in some patients.

In addition to the foregoing, previous studies have shown that extension movements cause an anterior migration of nuclear tissue, which conversely displaces posteriorly during flexion (Vanharanta et al., 1987). Therefore, the success of the extension principle of the McKenzie method may be linked to the ability of the exercises to have an effect on internal displacements and also reduce posterior protrusions in some intervertebral discs (Kopp et al., 1986). Alternatively, extension movements may relieve pain by reducing the forces acting on pain-sensitive tissues (Adams et al., 2000). Extension movements are hypothesized to unload the entire disc as the vertebrae can pivot around the apophyseal joints during the manoeuvre (Adams et al., 2000). Similarly, within the disc itself, extension causes a transfer of load from the anterior annulus and nucleus to the posterior annulus (Adams, 1994) and the effect is magnified after creep-loading (Adams, McNally, & Dolan, 1996). Sustained and repeated extension movements have been shown in some studies to increase the height of the spine presumably by unloading the disc and permitting rehydration (Magnusson, Simonsen, & Aagaard, 1996).

The findings of this study showed that there were no significant differences in the treatment outcome (mean change) between the CBMT and TBMT groups at the end of the 4^th^ and 8^th^ weeks of the study, except for vitality composite of the SF-12 where the TBMT group had significantly higher mean change. It was also observed that the CBMT had a higher mean change margin in the mental health domain of the SF-12 but was not statistically significant (p=0.053). The significant difference in vitality observed between both interventions may not be unconnected with the significant difference that existed in the baseline between both groups. In addition, the significant difference in the weight, with the app-based group being significantly heavier than the clinic-based group, may explain the subsequent difference in fatigue/vitality.

There are earlier claims that telerehabilitation enhances the psychological functioning of patients and their intrinsic motivation (Gale & Sultan, 2013; Irvine et al., 2015). There are specific mobile apps developed to enhance mental health of patients (Hind & Sibbald, 2015; Sagar & Pattanayak, 2015). As well, mobile apps developed for other therapeutic purposes have been reported to have significant effects on psychosocial health of patients (Blödt et al., 2014; Machado et al., 2016). Further studies are needed to confirm the effect TBMT on psychosocial constructs of patients from other populations.

Generally, there are more studies on the use of telerehabilitation to aid in the assessment of patients with LBP (Axén, Bergström, & Bodin, 2014; Palacín-Marín et al., 2013; Truter, Russell & Fary, 2014) than for treatment purposes. Telerehabilitation includes the use of smartphones, telemonitoring, mobile apps, and similar online tools and devices to educate patients, caregivers and health professionals about disease; to promote healthy living in the general public; and to provide an interactive platform to aid communication and feedback between individuals and those helping them manage their disease. These approaches have been reported to be effective in various patient populations. Systematic reviews of literature support the efficacy and effectiveness of telerehabilitation (Dahlia et al., 2013). However, there is still a paucity of evidence of clinical benefit from such technologies, thereby making it of research interest.

There is a wide range of heterogeneity between studies with respect to methodologies, population samples, clientele, settings, and outcomes measured. Many of the studies reported similar or better clinical outcomes for telerehabilitation when compared to conventional interventions (Man, Soong, Tam, & Hui-Chan, 2006), while no studies reported worse outcomes with telerehabilitation (Dahlia et al., 2013). Also, there is an abundance of commercially available applications offered for pain management. However, one of the major shortcomings of existing apps is that they rarely adhere to established guidelines or link to scientifically proven concepts (Abroms, Padmanabhan, Thaweethai, & Phillips, 2011; Huckvale, Car, Morrison, & Car, 2012), and there is only modest evidence for improvement in general health care based on smart phone app use. Vardeh, Edward, and Jamison (2013) submit that there are minimal data available to judge the efficacy of smartphone interventions for pain.

The McKenzie therapy approach propagates the principle of extension in the management of LBP and also advocates self-care. Although the efficacy of McKenzie method has been established by several studies, it is yet to be proven whether the method will produce similar results if self-administered outside the conventional clinic-based approach. The comparability in findings between the CBMT and TBMT at 4^th^ and 8^th^ week from this study, supports the assertion that mobile technology will not completely replace the traditional in-person interaction with a health-care professional (Vardeh, Edward & Jamison, 2013). However, the findings of this study seem to be consistent with the opinion that telerehabilitation is a viable link that may help remedy the challenges of barriers of distance, time, and travel to receive care (ATA, 2013). In particular, this study’s findings supports that TBMT may help improve access to the McKenzie methods. Considering that proper evaluation and appropriate treatment using the McKenzie therapy is premised on specialized training in the McKenzie Mechanical Diagnosis and Therapy (Clare, Adams, & Maher, 2004; Donelson, 1990; Miller & Herbowy, 2002; Simonsen, 1998).

## CONCLUSION

McKenzie extension protocol conducted via a telerehabilitation platform has comparable outcomes with clinic-based McKenzie therapy. Thus, telerehabilitation application of the McKenzie extension is effective in management of patients with chronic non-specific low-back pain. Hence, telerehabilitation-based McKenzie therapy may help bridge the gap in the non-availability of clinic-based McKenzie therapy facilities, especially in remote settings.

## Figures and Tables

**Table 1:** Independent t-test Comparison of the Participants’ General Characteristics by Treatment Groups

CBMT	TBMT	All participant			
Variable	(n = 26) χ̄ ± SD	(n = 21) χ̄ ± SD	t-cal	(n = 47) p-value	χ̄ ± SD
Age (y)	50.0 ± 10.7	47.3 ± 11.6	0.845	0.403	48.8 ± 11.1
Weight (Kg)	71.0 ± 7.84	79.1 ± 13.1	−2.639	0.011	74.6 ± 11.2
Height (m)	1.64 ± 0.08	1.68 ± 0.08	−1.644	0.107	1.66 ± 0.08
BMI (Kg/m^2^)	26.4 ± 3.42	27.9 ± 3.65	−1.447	0.155	27.1 ± 3.56
Pain duration (month)	8.31 ± 3.20	9.76 ± 2.70	−1.660	0.104	8.96 ± 3.04

Alpha level was set at p <0.05; *Key: CBMT = Clinic-Based McKenzie Therapy Group; TBMT = Telerehabilitation-Based McKenzie Therapy Group; x̄ = Mean; SD = Standard deviation*

**Table 2:** Participants’ profile based on the McKenzie Institute Lumbar Spine Assessment Format

Variable	CBMT (n=26) n(%)	TBMT (n=21) n(%)	χ^2^	p-value
Gender				
Male	6(23.1%)	7(33.3)	0.611	0.435
Female	20(76.9%)	14(66.7)		
Occupation				
Artisan	4(15.4%)	2(9.52%)	6.772	0.453
Trading	9(34.6%)	4(19.05%)		
Civil service	4(15.4%)	7(33.33%)		
Teaching	5(19.2%)	2(9.52%)		
Nursing	1(3.85%)	2(9.52 %)		
Student	0(0.0%)	1(4.76 %)		
Retiree	2(7.69%)	3(14.3%)		
Driver	1(3.85%)	0(0.00%)		
Reduced ROM				
Extension	1(3.85%)	0(0.00%)	3.719	0.293
Flexion	2(7.69%)	0(0.00%)		
Right side flexion	0(0.00%)	1(4.76%)		
Nil	23(88.5%)	20(95.2%)		
Cause				
Bending	2(7.69%)	1(4.56%)	10.251	0.114
Rigorous activity	8(30.8%)	0(0.00%)		
Lifting	2(7.69%)	3(14.3%)		
Poor posture	2(7.69%)	2(9.52%)		
Prolonged sitting	3(11.5%)	1(4.76%)		
Standing	1(3.85%)	2(9.52%)		
No apparent reason	8(30.8%)	12(57.1%)		
Aggravating factors				
Bending	9(34.6%)	6(28.5%)	1.867	0.867
Lying	1(3.85%)	0(0.00%)		
Sitting/rising	4(15.4%)	2(9.52%)		
Sitting	4(15.4%)	4(19.0%)		
Standing	4(15.4%)	5(23.8%)		
Walking	4(15.4%)	4(19.0%)		
Alleviating factors				
Lying	16(61.5%)	8(38.1%)	3.980	0.409
Sitting	6(23.1%)	9(42.9%)		
Standing	1(3.84%)	1(4.76%)		
Walking	3(11.5%)	2(9.52%)		
Altering assumed positions	0(0.00%)	1((4.76%)		
LBP disturbs sleep				
Yes	13(50.0%)	13(61.9%)	0.666	0.414
No	13(50.0%)	8(38.1%)		

Key: ROM = Range of Motion; % = Percentage; CBMT = Clinic-Based McKenzie Therapy; TBMT = Telerehabilitation-Based McKenzie Therapy

**Table 3:** Comparison of the Participants’ Baseline Parameters

Variable	CBMT (n=26)	TBMT (n=21)	Statistics	p-value
Parametric variable†	χ̄±SD	χ̄±SD	t	
Pain intensity				
Current	5.00 ± 1.96	4.29 ± 1.38	1.409	0.166
Average	5.31 ± 1.44	4.95 ± 0.92	0.982	0.332
Least	2.62 ± 1.27	2.76 ± 1.41	−0.375	0.710
Worst	7.08 ± 1.41	7.24 ± 1.00	0.442	0.661
QVAS score	58.0± 14.1	54.9 ± 8.67	0.863	0.393
Activity limitation	11.8 ± 4.78	10.2 ± 4.66	1.133	0.263
Back muscles endurance	20.4 ±12.8	25.8 ± 15.2	−1.319	0.194
General health status				
Scale				
Physical function	26.3±22.7	30.9±24.3	0.979	0.500
Role Limitation-physical	97.3± 9.51	93.3± 14.1	1.151	0.256
Bodily Pain	58.7± 24.1	67.7±16.6	1.447	0.155
Health Perception	40.4± 26.0	38.3± 17.4	0.310	0.758
Energy/Vitality	41.5± 23.3	61.0± 23.2	2.846	0.007
Social Functioning	90.4± 12.4	83.3±12.1	1.960	0.056
Role Limitation – emotional	94.6± 12.8	86.7±17.4	1.798	0.079
Mental Health	53.1± 13.7	58.2± 15.3	1.213	0.231
Domain				
Mental Health	69.9±8.71	72.3 ± 5.74	1.080	0.286
Physical Health	64.2± 10.4	65.9±4.68	0.701	0.487
Non-Parametric variable‡	Mean rank	Mean rank	U	
Participation restriction	24.1	23.9	271.500	0.974

Key: χ̄ = Mean; SD = Standard Deviation; QVAS = Quadruple Visual Analogue Scale

† Parametric test - Independent t-test;

‡ Non-Parametric test - Mann-Whitney U

**Table 4:** Comparisons of Treatment Outcomes among Participants in CBMT across the Three Time Points of the Study (n=26)

Variable	Baseline	4^th^ week	8^th^ week	Statistics	p-value
Parametric variable†	χ̄±SD	χ̄±SD	χ̄±SD	F	
Pain intensity					
Current	5.00 ± 1.96^a^	3.42 ± 0.86^b^	1.54 ± 1.24^c^	58.798	0.001
Average	5.31 ± 1.44^a^	3.46 ± 1.07^b^	1.77 ± 0.91^c^	139.21	0.001
Least	2.62 ± 1.27^a^	1.23 ± 1.07^b^	0.35 ± 0.69^c^	85.571	0.001
Worst	7.08 ± 1.41^a^	4.58 ± 1.17^b^	3.04 ± 1.08^c^	163.188	0.001
QVAS score	57.9 ± 14.0^a^	38.2 ± 8.12^b^	21.2 ± 9.66^c^	138.715	0.001
Activity limitation	11.8 ± 4.78^a^	6.38 ± 3.02^b^	2.50 ± 1.72^c^	125.265	0.001
Back muscles endurance	20.4 ± 12.8^a^	29.1 ± 12.8^a^	35.4 ± 11.4^b^	101.397	0.001
General health status					
Scale					
Physical Functioning	22.3±22.7	58.3±21.2	69.9±18.3	76.455	0.001
Role Limitation-physical	97.3 ±9.51^a^	77.1 ± 9.00^b^	89.2 ± 16.5^c^	7.500	0.011
Bodily Pain	58.7 ± 24.1^a^	79.0 ± 13.5^b^	89.5 ± 14.5^b^	26.543	0.001
Health Perception	40.4 ± 26.0^a^	71.0 ± 26.0a	82.7 ± 14.1	67.957	0.001
Energy/Vitality	94.6 ± 12.8^a^	70.4 ± 12.8^a^	69.0 ± 11.4^b^	67.857	0.001
Social Functioning	90.4 ± 12.4^a^	78.8 ± 9.20 ^b^	76.9 ± 6.79^b^	29.167	0.001
Role Limitation-emotional	94.6 ± 12.9^a^	70.4 ± 12.9^b^	69.0± 11.4	67.857	0.001
Mental Health	70.8 ±13.7^a^	65.9 ± 13.6^b^	70.8 ± 4.25^c^	37.559	0.001
Domain					
Mental Health	69.9 ± 8.70^a^	71.9 ± 7.38^b^	75.9 ± 6.42^c^	11.747	0.001
Physical Health	64.2 ± 10.4^a^	72.4 ± 7.32^a^	80.1 ± 7.04^b^	41.684	0.001
Non-parametric variable‡	Mean rank	Mean rank	Mean rank	χ^2^	
Participation restriction	24.1^a^	26.1^b^	25.0^c^	59.769	0.001

† Parametric test - ANOVA (F-ratio) and LSD post-hoc multiple comparison;

‡ Non-Parametric test - Friedman’s ANOVA (χ^2^) and Wilcoxon signed ranked test

*Superscripts (^a,b,c^).* For a particular variable, mean values with different superscript are significantly (p<0.05) different. Mean values with same superscripts are not significantly (p>0.05) different. The pair of mean values that are significantly different have different superscripts assigned to them.

Key: χ̄ = Mean; SD = Standard Deviation; QVAS = Quadruple Visual Analogue Scale

**Table 5:** Comparisons of Treatment Outcomes among Participants in TBMT across the Three Time Points of the Study (n=21)

	Baseline	4^th^ week	8^th^ week		

Variable	χ̄±SD	χ̄±SD	χ̄±SD	Statistics	p-value
Parametric variable†	χ̄±SD	χ̄±SD	χ̄±SD	F	
Pain intensity					
Current	4.29 ± 1.38^a^	2.43± 1.25^b^	0.48 ± 0.51^c^	183.381	0.001
Average	4.95 ± 0.92^a^	3.00 ± 0.71^b^	0.76± 0.94^c^	317.377	0.001
Least	2.76 ± 1.41^a^	0.67 ± 0.91^b^	0.10 ± 0.30^c^	70.000	0.001
Worst	7.24 ± 1.00^a^	4.48 ± 1.21^b^	1.95 ± 1.56^c^	243.018	0.001
QVAS score	54.9 ± 8.67^a^	33.0 ± 6.74^b^	10.6 ± 7.86^c^	521.024	0.001
Activity limitation	10.2 ± 4.66^a^	5.38 ± 3.14^b^	2.29 ± 2.47^c^	78.362	0.001
Back muscles endurance	25.8 ± 15.2^a^	35.5 ± 15.0^a^	40.1 ± 13.6^b^	97.815	0.001
General health status					
Scale					
Physical Functioning	30.9 ± 24.3^a^	61.1 ± 21.3^b^	74.6 ± 16.3^c^	58.852	0.001
Role Limitation – Physical	25.8 ± 14.1^a^	83.3 ± 17.9b	90.0 ± 16.2^c^	0.488	0.493
Bodily Pain	67.6 ± 16.6^a^	76.2 ± 22.7	89.5 ± 10.9^c^	39.331	0.001
Health Perception	38.3 ± 17.5^a^	74.3 ± 16.6^b^	78.2 ± 16.3^c^	60.235	0.001
Energy/Vitality	60.9 ± 23.2^a^	81.9 ± 18.9^b^	82.9 ± 19.3^b^	12.050	0.002
Social Functioning	83.3 ± 12.1^a^	77.4 ± 7.52^b^	75.0 ± 10.3^b^	10.000	0.005
Role Limitation –Emotional	86.7 ± 17.4^a^	66.7 ± 7.64^b^	66.7 ± 10.3^b^	26.667	0.001
Mental Health	58.2 ± 15.3^a^	66.0 ± 7.23^b^	68.3 ± 5.12^c^	9.510	0.006
Domain					
Mental Health	72.3 ± 5.73^a^	73.0 ± 4.64^b^	73.2 ± 4.46^c^	9.510	0.006
Physical Health	65.8 ± 4.68^a^	74.4 ± 7.48^b^	79.5 ± 6.48^c^	70.028	0.001
Non-parametric variable‡	Mean rank	Mean rank	Mean rank	χ^2^	
Participation restriction	23.9^a^	21.4^b^	22.8^c^	131.236	0.001

† Parametric test - ANOVA (F-ratio) and LSD post-hoc multiple comparison;

‡ Non-Parametric test - Friedman’s ANOVA (χ^2^) and Wilcoxon signed ranked test

*Superscripts (^a,b,c^).* For a particular variable, mean values with different superscript are significantly (p<0.05) different. Mean values with same superscripts are not significantly (p>0.05) different. The pair of mean values that are significantly different have different superscripts assigned to them.

Key: χ̄ = Mean; SD = Standard Deviation; QVAS = Quadruple Visual Analogue Scale

**Table 6:** Comparison of Participants’ Treatment Outcomes (Mean Change) for the Continuous Variables at Week 4 of the Study

Variable	CBMT (n=26) χ̄±SD	TBMT (n=21) χ̄±SD	Statistics	p-value
Parametric variable†	χ̄±SD	χ̄±SD	t	
Pain intensity				
Current	1.58 ± 1.50	1.86 ± 0.79	−0.772	0.444
Average	1.85 ± 1.43	1.95 ± 0.74	−0.308	0.760
Least	1.39 ± 0.98	2.10 ± 1.55	−1.915	0.062
Worst	2.50 ± 1.55	2.76 ± 1.34	−0.610	0.545
QVAS score	19.7± 12.3	21.9± 7.57	−0.706	0.484
Activity limitation	5.42 ± 3.04	4.86 ± 4.53	0.511	0.612
Back muscles endurance	8.69 ± 5.73	9.71 ± 6.38	−0.578	0.566
General health status				
Scale				
Physical Functioning	32.0 ± 27.9	30.1 ± 31.9	0.217	0.829
Role Limitation - Physical	20.2 ± 17.6	10.0 ± 22.5	1.741	0.089
Bodily Pain	20.3 ± 22.5	8.57 ± 25.5	**1.686**	**0.099**
Health Perception	30.6 ± 37.2	35.9 ± 25.2	0.565	0.575
Energy/Vitality	30.7 ± 23.5	20.9 ± 27.9	1.310	0.197
Social Functioning	11.5 ± 12.7	5.95 ± 10.9	1.594	0.118
Role Limitation – Emotional	24.2 ± 19.2	20.0 ± 17.7	0.776	0.442
Mental Health	12.9 ± 14.4	7.82 ± 17.3	**1.084**	**0.284**
Domain				
Mental Health	1.95 ± 7.92	0.71 ± 7.46	0.552	0.584
Physical Health	8.26 ± 10.2	8.52 ± 10.0	0.090	0.929
Non Parametric variable‡	Mean rank	Mean rank	U	
Participation restriction	26.4	21.0	210.000	0.176

Key: χ̄ = Mean; SD = Standard Deviation; QVAS = Quadruple Visual Analogue Scale

† Parametric test - Independent t-test;

‡ Non-Parametric test - Mann-Whitney U test

**Table 7:** Comparison of Participants’ Treatment Outcomes (Mean Change – i.e., Week Eight minus Baseline) for the Continuous Variables at Week 8 of the Study

Variable	CBMT (n=26) χ̄±SD	TBMT (n=21) χ̄±SD	Statistics	p-value
Parametric variable†	χ̄±SD	χ̄±SD	t	
Pain intensity				
Current	3.46± 2.30	3.89 ± 1.29	0.618	0.540
Average	3.53 ± 1.52	4.19 ± 1.08	0.982	0.332
Least	2.27 ± 1.25	2.67 ± 1.46	−0.375	0.710
Worst	4.04 ± 1.61	5.29 ± 1.55	−0.442	0.661
QVAS score	36.8 ± 15.9	44.3 ± 8.89	0.863	0.393
Activity limitation	9.31 ± 4.24	7.95 ± 4.12	1.104	0.276
Back muscles endurance	15.0 ± 7.58	14.3 ± 6.62	0.321	0.749
General health status				
Scale				
Physical Functioning	43.6 ± 25.4	43.7 ± 26.1	0.008	0.994
Role Limitation - Physical	8.08 ± 15.0	3.33 ± 21.9	0.879	0.384
Bodily Pain	27.3 ± 27.0	21.9 ± 16.0	0.808	0.423
Health Perception	42.3 ± 32.5	39.8 ± 23.5	0.301	0.765
Energy/Vitality	45.4 ± 31.3	21.9 ± 28.9	2.646	0.011
Social Functioning	13.5 ± 12.7	8.33 ± 12.1	1.406	0.167
Role Limitation - Emotional	25.6 ± 15.8	20.0 ± 17.7	1.137	0.261
Mental Health	17.7 ± 14.7	10.0 ± 14.9	**1.759**	**0.085**
Domain				
Mental Health	6.01 ± 8.94	0.901 ± 8.48	**1.991**	**0.053**
Physical Health	15.9 ± 12.6	13.7 ± 7.47	0.730	0.469
Non Parametric variable‡	Mean rank	Mean rank	U	
Participation restriction	25.6	22.1	232.000	0.380

Key: x̄ = Mean; SD = Standard Deviation; QVAS = Quadruple Visual Analogue Scale

† Parametric test - Independent t-test;

‡ Non-Parametric - Mann-Whitney U test
